# Experiences of Animal Loss and Grief among Zoo Professionals and Volunteers: A Qualitative Study

**DOI:** 10.3390/ani14202925

**Published:** 2024-10-11

**Authors:** Jennifer Currin-McCulloch, Nichole Louise Nageotte, Abigail Walker, Shelby McDonald, Lori Kogan

**Affiliations:** 1 School of Social Work, Colorado State University, Fort Collins, CO 80523, USA; abby.walker@colostate.edu (A.W.); shelby.e.mcdonald@colostate.edu (S.M.); 2 Department of Community Research & Evaluation, Denver Zoo Conservation Alliance, Denver, CO 80205, USA; nnageotte@denverzoo.org; 3 Clinical Sciences, Colorado State University, Fort Collins, CO 80523, USA; lori.kogan@colostate.edu

**Keywords:** grief, loss, zoos, zoo animals, mental health, conservation

## Abstract

**Simple Summary:**

Zoo professionals and volunteers care for numerous animals, creating the potential for exposure to multiple deaths and transfer-related losses. Despite this, there is limited research exploring how zoo professionals and volunteers experience and adapt to zoo animal loss. This qualitative study was designed to explore the personal experiences of zoo animal loss among zoo professionals and volunteers, including their emotional challenges, coping mechanisms, and support-related needs. We found participants’ responses highlighted two main themes: the lasting toll of animal-related loss and their interpersonal experience of loss. Within these themes, zoo professionals and volunteers’ responses provided insights into experiences related to deaths (both expected and unexpected), transfer losses, experiences of complicated grief, and interpersonal factors that interfered with their ability to process their loss. Participants’ suggestions for support when faced with these experiences included enhanced communication before pending losses and following deaths and transfers, environments conducive for memorializing rituals, and increased access to grief support resources.

**Abstract:**

Zoo professionals and volunteers play essential roles in the wellbeing and conservation of a diverse array of animal species. Despite the growing body of literature revealing the psychological impacts of pet loss, there remains a dearth of information describing the experience of animal loss among those who work in zoo settings. This qualitative study explored the personal experiences of zoo animal loss among volunteers (*n* = 12), animal care and health professionals (ACHPs) (*n* = 135), and other zoo staff (*n* = 35) who participated in a larger mixed-method study. Participants responded to five open-ended questions exploring their most significant zoo animal death loss, where or from whom they found the most support, how the zoo community could better support them, advice for zoo leaders, and other thoughts about their grief and animal-related loss experiences. Using thematic analysis, two key themes were identified: the lasting toll of zoo animal loss and zoo professionals’ and volunteers’ interpersonal experiences. Participants described their experiences with animal transfers and both expected and unexpected deaths. Requests for support focused on better communication, grief resources, and opportunities to recognize and mourn animal losses. These findings suggest that zoo animal loss can negatively impact zoo professionals’ and volunteers’ psychological health.

## 1. Introduction

Humans form close emotional attachments with a wide array of animals, including domesticated, wild, and feral species [1]. For example, most pet owners view their pets as family members [2,3,4], and numerous studies report that the attachment and connection humans feel toward their pets parallels bonds between people [5,6,7,8,9,10,11,12]. Consequently, over the past few decades, there has been substantial growth in research examining human responses to pet loss [6,9,13,14,15,16,17]. These studies suggest that the death of a pet often results in significant grief, marked by intense feelings of loss and sadness [18,19,20,21], emulating the grief experienced following the loss of a human companion [5,8,19,22,23,24,25]. Despite these similarities, the death of a pet typically does not involve traditional funeral rituals, thereby limiting how owners are able to express and share their grief [26,27,28,29,30]. This often results in “disenfranchised” or unrecognized grief, leaving pet owners to cope with their loss without the support of friends, family, or the broader community [31,32,33,34,35].

Despite the growing body of literature pertaining to the psychological impacts of pet loss, there remains a dearth of information pertaining to the impact of animal loss experienced by those who work with animals. This study addresses this gap by qualitatively exploring the emotional challenges, coping mechanisms, and support-related needs of zoo professionals and volunteers when dealing with the loss of zoo animals through death or transfer.

### 1.1. Emotional Challenges for Zoo Professionals

Zoo professionals and volunteers play essential roles in the wellbeing and conservation of a diverse array of animal species. A natural part of these zoo positions involves animal loss via death, animal transfer, or a change in job duties [36]. These losses, including the witnessing of end-of-life processes, such as euthanasia, can have profound psychological impacts [37,38,39,40,41]. This emotional burden may be further intensified when caring for endangered species, as professionals and volunteers are often acutely aware of their critical roles in species survival and conservation efforts [42,43,44,45,46]. These losses can further be complicated by the need to continue their occupational responsibilities as well as the public visibility of their work [47,48].

Additionally, the diverse range of species in zoos necessitates that zoo professionals cope with varying lifespans and death experiences. Some species, such as elephants, certain reptiles, and birds, may live for many decades, offering the opportunity for caregivers to build long-term bonds [47,49,50]. Other species have much shorter lifespans, leading to more frequent experiences of loss. This variability can make it challenging for zoo professionals to navigate their grief and can create a constant cycle of attachment and loss that requires ongoing emotional resilience and robust support mechanisms [36,51].

Recent research suggests that those who work closely with animals in zoo settings are likely to frequently encounter complex and challenging situations involving the loss and/or death of the animals under their care [47,52,53]. For instance, a recent study of zoo professionals found that in the previous 12 months, 80% of the zoo professionals experienced at least one unexpected death and 74% experienced anticipated loss, with more than 50% reporting that these losses had occurred within the past three months [36]. Prior non-empirical work argues that these animal loss experiences negatively impact zoo professionals’ and volunteers’ wellbeing, contributing to feelings of guilt, sadness, and grief [52]. Furthermore, McDonald et al. [36] found that stronger bonds with those animals lost (through death, transfer, etc.) were associated with higher levels of depression and anxiety. These findings highlight the need for a deeper understanding of animal bonds and loss and death experiences in the zoo industry and the importance for considering the impacts of these potentially adverse experiences on the emotional, psychological, and professional wellbeing of these professionals.

Despite the fact that prior research has suggested that stronger bonds are associated with higher levels of mental health symptomatology among animal care and health professionals (ACHPs), an emerging body of research suggests that strong bonds and positive relationships with animals should be fostered and encouraged within zoo settings. Indeed, there is evidence that the quality of bonds with zoo animals (e.g., time in interacting with, soliciting contact with, and talking to them) is associated with a variety of positive outcomes for zoo animals, such as reproductive success in small cat species [54] and lower fecal glucocorticoids among white rhinoceroses [55] and clouded leopards [56]. Relatedly, prior research demonstrates that good keeper–elephant relationships and bonds are beneficial to both humans and elephants [49]. Thus, it is important to determine how the zoo industry can nurture and encourage healthy bonds between zoo professionals/volunteers and zoo animals while simultaneously creating a culture and offering supportive services that offer zoo professionals and volunteers the tools and resources they need to handle grief and loss in healthy, sustainable ways.

### 1.2. Current Study

Before the zoo industry can identify macro-level sustainable structural changes that can address the occupational challenges resulting from animal transfer and death, a better understanding of the individual experience of these unique occupational stressors is needed. Decades of research have found that zoo professionals’ job-related experiences have direct and indirect influences on their mental health and wellbeing and that work-related stressors can increase the risk of development or exacerbation of mental health problems [57,58]. This knowledge has led to a plethora of work-related interventions that focus on the individual while often ignoring larger, more systemic influences [59] that may limit their effectiveness [60,61]. Therefore, it is important, when assessing work stressors and potential interventions, to integrate multiple levels of analysis (micro and macro) to gather a thorough understanding of interrelated individual and systemic factors, which may be essential for the success of intervention strategies [59,62]. Our qualitative study was designed to better understand the micro-level loss-related stressors experienced by U.S. zoo professionals and volunteers. This knowledge will lay the foundation for understanding animal-loss-related stressors and buffers within the zoo industry [52,63,64]. To this end, this study was designed to better understand the grief- and loss-related stressors experienced by zoo professionals/volunteers at the personal/micro level and help to identify macro/systemic work-related support found to be of help when coping with zoo-animal-related loss.

## 2. Methods and Materials

### 2.1. Positionality Statement

While designing this research study, we developed protocols to help us to reflect and process how our professional and personal identities and experiences could influence each phase of the research study. J.C.-M. is an academic researcher with 20 years of social work practice working alongside those experiencing life-limiting illnesses and their care partners; N.N. is a zoo social science researcher with a background in zookeeping, informal science learning, and conservation behavioral change. A.W. is a social work graduate student and research assistant; S.M. is an academic researcher who focuses on human–animal interactions and was previously employed as a researcher in a zoo setting; and L.K. is an academic researcher who focuses on human–animal interactions.

### 2.2. Study Design

This qualitative research was a part of a larger mixed-method study that explored U.S. zoo professionals’ and volunteers’ experiences of anxiety, depression, burnout, professional fulfillment, and animal-related loss. In this larger study, participants completed an anonymous online survey with both qualitative and quantitative items [36].

The qualitative portion of the survey consisted of the following five questions:

1. In thinking back on your time at the zoo, please describe the most significant death loss you have experienced with an animal at the zoo.
2. What and/or who offered you the most support in coping with the death of animals at your zoo?
3. Please describe how your zoo community could better support you in coping with animal-related losses (e.g., animal transfers and deaths) at the zoo.
4. If you could give one piece of advice for leaders at the zoo in how best to support you leading up to or after the loss of an animal at the zoo, what would you say?
5. Please share other thoughts that you have in reflecting on your experiences of grief and animal-related loss in your role at the zoo.

Online network sampling was used to recruit participants from professional association listservs (AZA, etc.) and social media platforms. Surveys were completed electronically (on the Alchemer survey platform) between July and October 2023. The average time for the survey completion was 15 min.

We integrated the following statement into the informed consent document to acknowledge potential risks that participants may experience when recounting their experiences of zoo animal loss and to offer a grief support resource:

The survey includes some questions pertaining to feelings of loss and grief surrounding the death or transfer of zoo animals. If you experience any emotional discomfort from taking the survey, you can contact your zoo’s human resource department to access your mental health benefits or contact Lap of Love https://www.lapoflove.com/ (accessed on 5 May 2024)—they offer free support for animal loss and grief.

### 2.3. Participants

Surveys were completed by 1695 individuals, although not all the individuals provided responses to each qualitative item. For the larger sample, most participants identified as white (91%) and female, feminine, or woman (79%). Around 20% identified as LGBTQIA+, and the mean age was 37. Participants were provided with a list of 19 zoo roles from which they could identify their staff or volunteer role. Participants were assigned to one of three groups based on the role they selected: Volunteer, ACHPs, and Other Staff. ACHPs included individuals who were directly responsible for the care and wellness of animals, such as zookeepers, curators, veterinarians, veterinary technicians, and nutritionists. The “Other Staff” category included all the paid staff not directly responsible for the care and wellbeing of animals, such as individuals in education, marketing, or guest service roles. For the current qualitative study, 10% of the participants from each group were randomly selected using IBM SPSS, version 28. This resulted in a total sample of 182 participants (12 Volunteers, 135 ACHP, and 35 Other) for qualitative analysis. Participants were only included in the random sample if they provided an answer to each of the five qualitative questions. If participants were discarded from the analysis for not answering at least one of the five questions, additional participants were randomly selected until 10% of each group was represented.

### 2.4. Analysis Methods

This study used the applied thematic analysis method [65] to inductively analyze participants’ responses to the survey’s five open-ended questions. Thematic analysis requires that researchers approach data without predetermined themes and instead, through the course of the analysis, determine themes based on participants’ stated experience with a specific phenomenon.

A team of three (J.C.-M., N.N., and A.W.) participated in the thematic analysis. We analyzed the participants’ responses to each open-ended survey question similarly: (1) individually read each response and created initial codes to represent the experiences of zoo animal loss and grief; (2) met as a team to determine codes, coding definitions, and themes; (3) addressed disparities in coding until a coding consensus was reached; (4) developed a codebook to support the application of the themes across the entire data set; and (5) selected salient quotes to represent each theme. To protect the identities of the study participants, we intentionally removed any identifying information from the participants’ quotes, including only their study identification number and the category of their role (e.g., Volunteer, ACHP, or Other Staff). Given the traumatic nature of many of the losses described and the potential for activating responses from the readers, we intentionally chose to limit shared quotes to those that reflect the least-graphic details of the death and post-death care of the animals.

### 2.5. Trustworthiness

We integrated several processes to enhance trustworthiness in the data analysis. Overall, we met 15 times during the data analysis phase to review the data, line by line, and to develop the codebook structure. We also created an audit trail of memos to track thematic and analytic decision-making processes within team meetings. Examples of memos included descriptions of code names, definitions and examples of how to apply codes, as well as memos to follow the development and adaptation of themes, and the emergence of code and meaning saturation [66]. Incorporating three coders with differing personal and professional experiences in grief and loss and zoo settings provided rich perspectives from which to interpret the data and develop implications for enhancing the mental wellbeing of zoo professionals and volunteers. Microsoft Excel was utilized to organize individual codes and integrate team codes into the final codebook structure.

## 3. Results

The results of the thematic analysis identified two key themes, with several sub-themes, within the participants’ descriptions of their zoo animal loss and grief: the lasting toll of animal loss and the interpersonal experience of loss.

### 3.1. The Lasting Toll of Animal Loss

The zoo professionals and volunteers provided vivid descriptions of their encounters with animal loss through the death or transfer of zoo animals. They portrayed experiences leading up to their most significant losses, details of the transfer or death, their emotional responses to the loss, and organizational and community responses to these losses. Although asked to share about the most significant loss, it was not uncommon for participants to share several losses that had lasting impacts. The following quote by an ACHP provides a glimpse into the nuanced ways that animal loss can emerge over the course of one’s career:

It is difficult to quantify [the most significant loss]. They are not all the same, and each one has different qualities that make them unique. The ones that stick out are the first traumatic death I witnessed (an accidental cut of an [animal’s] artery and watching it bleed out), deaths of animals with whom I had extremely close relationships, deaths of baby animals, and deaths that felt like they could’ve been avoided/prevented, unexpected and/or traumatic deaths, and the rapid repetition of losses.

(P381, ACHP)

Categories of losses that emerged throughout the participants’ responses included animal transfers and expected deaths due to older age or a life-limiting illness and those due to an unexpected event resulting from an accident, medical procedure, or predation. Within these main categories of loss, other characteristics appeared to raise the level of the significance of the loss, such as the deaths of charismatic animals, paired animals (e.g., a newborn animal and their parent or highly bonded animals), and ambassador animals (those used to educate the public about the species). For example, an ACHP described the death of an animal for whom they cared deeply:

She was older, but we watched as she got worse and worse. It was hard. I held her as she died, and she looked at me the whole time as if for comfort. It’s a lot to deal with, no matter how small of an animal. You’re their world and their day to day.

(P532, ACHP)

This quote exemplifies the depth of feelings many zoo professionals and volunteers expressed when describing the deaths of animals with whom they shared close bonds.

Despite the focus of the survey to gather participants’ experiences about their most significant death loss, many participants chose to share about losses related to animal transfers to other facilities. A staff member summarized what many felt regarding discrepancies in the culture around animal death and transfer losses: “I think we focus a lot on animal death as a loss but not the animal transfers. I think we could do better in preparing our teams for this and what they see on social media after this animal is moved (good or bad)” (P1249, other staff). Descriptions of organizational culture surrounding transfers varied: Although some participants reported a positive organizational culture around transfers, many others described organizations with more veiled transfer cultures. Positive transfer descriptions often focused on zoos where zoo professionals and volunteers were kept abreast of pending moves and had time to create a meaningful parting with an animal—to say goodbye. However, more commonly, participants shared proclamations, such as, “It seems to happen very quickly, or we do not find out until their pre-shipment exam” (P1374, ACHP). Participants also expressed frustration in having limited say in the care of animals before or during transfers and received limited communication from the new facility. Despite the common perception that well-defined structures to support open communication around transfer processes were lacking, many participants reported feeling that the move of animals to other facilities offered hope that these animals might find better pairings or that peer facilities could help to create more positive futures for dwindling species.

In terms of death losses, the participants’ responses suggest that the circumstances leading up to the death and the participants’ self-determined levels of personal responsibility in that death are influential in their grief. Regardless of whether the individual felt they had a clear role in the animal’s death, expressions of guilt emerged surrounding actions leading up to the animal’s death. Not being present when an animal died frequently resulted in participants holding guilt. For instance, an ACHP shared when they found an animal lifeless as they entered the zoo habitat in the morning: “I found a [primate] dead from the plague. She was a little off when I left the night before and then dead the next morning. I felt guilty for not recognizing that she was more ill than I thought” (P179, ACHP). Often, the participants’ narratives reflected remorse in not being a stronger advocate for an animal:

[My] most significant [loss] was a female we lost after five procedures in two weeks, and she was on critical care—a full-time catheter. The original exam under anesthesia did not need to be done. We lost her to starvation because she was not eating (too many exams without time for her to recover and start eating again). This happened over three years ago. Every time I think about it, I cry and feel guilty that I did not speak up enough that what I felt we were doing to her was wrong.

(P82, ACHP)

Others believed that the care they provided directly led to the animal’s death:

I’m not good enough in my role. Last week our [mammal] became acutely ill and died. She did not respond to any of my treatments. I feel like I failed her and like I am not a good-enough vet. This is the hardest loss I have ever had aside from my own pets.

(P43, ACHP)

As exemplified in the above quote, feelings of guilt often were associated with complicated grief responses that lasted from months to several years.

Secondary losses often appeared as a result of animal transfer or death and added layers of complexity in how zoo professionals and volunteers adapted to the initial animal loss. The participants shared stories of walking by specific environments and missing that special animal—someone to talk with, whistle for, or share moments of deep affection. If animals died while participants were not at work, coming back for the first time after losing an animal created emotional distress: “It was very difficult coming back to work after those days and seeing an empty stall/not hearing her call/and not having that special animal anymore to comfort” (P1487, ACHP). Another participant shared, “I cried for days and didn’t want to come to work knowing I would not see her” (P2293, ACHP). In addition, secondary losses associated with entire flocks or herds lost because of illness, tragedies, or dying out included a pending loss of their role, ability to work with specific animals, or their career at their facility.

#### Individualized Adaptation to Loss

The zoo professionals and volunteers shared their life experiences with loss, personal coping strategies, and access to resources, which helped to buffer their individual responses to loss. Years of experience in the field created more exposure to loss and, for some, resulted in a mindset that grief would be commonplace in their career. Working for years in the field led one zoo professionals to proclaim, “I’m a long-time professional; animal deaths are a part of the job and have little to no impact on my negative feelings of the industry. Loss is difficult but an expectation when working with any living thing” (P838, ACHP). For others, the compounded loss appeared to create what they described as “numbness”, “being withdrawn”, or a “blocking out of feelings”. They portrayed how these experiences impacted their own and coworkers’ interpretations of their work with animals: “I think, over the years, with each new loss, I have started to become more-and-more numb to loss. Something along the lines of compassion fatigue but not quite, I guess” (P1547, ACHP). Another explained, “Birdkeepers are often thought [to be] cold hearted because we experience loss often, but it doesn’t hurt any less, we just become numb to it” (P827, ACHP). Numbing or dissociating from the loss appears to be one way some participants protect themselves from feeling their pain:

This year has been a really hard one for my department. We’ve lost almost one animal per month, and each new one has left me feeling more numb than the last. I think it’s caused me to close up a little bit to try to avoid feeling so much pain.

(P1350, Other Staff)

Resonating among participants’ statements was the desire for systems to do better in developing interpersonal and organizational approaches to help to ameliorate the pain resulting from continued exposure to animal loss.

Emotional responses to zoo animal loss varied by each individual’s description of their unique experience, as well as by the time in their career, views about death, and access to social support. Words used to characterize their emotional state included “shock”, “traumatized”, “burned out”, “isolated”, “grateful”, and “at peace”. Experiences of isolation around their loss presented additional barriers for processing their grief. Several participants noted that their zoos chose not to share about deaths with the public and some prohibited zoo professionals from even sharing about the deaths with their friends and family. The following quote highlights this disenfranchised grieving process:

No animal death is ever reported to the public, due to the fear of a backlash, but it hurts. We want to mourn publicly, but we’re not allowed to. Not even on our own social media. I miss my sweet boy, and I am crying writing this because it’s the most I’ve been able to write to anyone.

(P548, ACHP)

The participants’ narratives also indicated that, for some, external communication with the public had benefits in creating community and a social sanctioning of the loss. In these cases, it also helped to reduce the experiences of volunteers and zoo professionals having “wounds reopened” in needing to explain to inquiring guests about why their favorite animal wasn’t in its habitat.

### 3.2. The Interpersonal Experience of Loss

The zoo professionals’ and volunteers’ responses described interpersonal dynamics, which they felt had an influence on their ability to process their loss. These included interpersonal reactions and communication leading up to and following a loss, decisions about work tasks, and interpersonal trust.

One commonly expressed sentiment among the participants involved tensions or differences with members or leaders of their organization. These challenging dynamics were described by the participants as involving poor communication, lack of trust in individual expertise, staffing shortages, and behaviors that seemingly prioritized finances or the zoo’s public image. Stories of delays in treatment led to the participants witnessing animals’ extended suffering. They portrayed this suffering as unnecessary and futile: “The hardest for me, personally, in a professional setting was the argument between three staff members over [an animal] who was suffering from a leg infection/leg cancer and the delay it caused in euthanasia, extending the animal’s suffering” (P480, ACHP). These encounters often led to issues of trust among zoo professionals across professional roles:

It was a loss after I had disagreed with the decision to do the procedure, and she died from a complication from the unnecessary procedure. It changed a lot about how I make decisions around vet care and had led to a lot of trust issues with the vet team at the time.

(P77, ACHP)

Decisions to exclude zoo professionals from discussions about euthanasia for animals with whom they worked closely caused distressing grief impacts. For example, one staff member recounted:

I came to work one day and was told that [a charismatic animal] was being put down. I had not been included in any discussions about this and was blindsided [and] then required to attend the takedown. No time to spend with him prior to euthanasia, no time to grieve, just told to keep guests away. It was a miserable day. He was a friendly, loving [being] and great to interact with. He knew my voice and whistle and came when I called him. It was a hard loss.

(P353, Other Staff)

In addition to the psychological impact, not allowing ACHPs time to process following an animal’s death can become a safety concern when these individuals have to remove an animal’s body from a large-animal environment while still in a state of shock.

Frustrations were also voiced by the participants surrounding organizational decisions around staffing, which led to deaths that the participants felt may have been avoided or caused less trauma for the animal, zoo staff, volunteers, and guests. An animal care worker described their experience when an animal went into distress before all the necessary staff arrived for the day:

The individual [animal] died on the table, with guests watching through the viewing windows, which felt like an invasion of a private moment. This death hit me the hardest because I wondered if there had been vet techs on site at the time, if they could have saved him where we failed. Necropsy revealed that there was nothing we could have done in the long term, but the way that he died was traumatic for all involved.

(P1015, ACHP)

In some cases, leadership decisions interfered with the participants’ ability to grieve on their own terms. The zoo professionals reported having to continue working when they had recently experienced a significant loss and feeling like “the show must go on” (P2192, ACHP). The zoo professionals also had to prepare the body for disposal following the death, in ways that sometimes entailed graphic dismemberment for organ retrieval or to fit into freezer storage.

#### 3.2.1. Feeling Left Out of Communication

The participants shared that communication policies, both pre- and post-loss, were often influential on their ability to adapt to their loss. The participants expressed a desire to be informed of decisions related to pending transfers and end-of-life decisions, including euthanasia. This was especially important for the ACHPs responsible for the day-to-day care and health of the animals:

There have been many times where the animal under my care, that’s in decline, will have an emergency. I will notify a supervisor, and they will pick up the animal to bring to the vet, or I will bring them and then I will have to leave while they work on the animal and then no one tells me that the animal passed, or it was decided to euthanize it. I have stopped by our animal hospital for an update hours later, and they will be surprised no one told me that the animal had passed. It’s not that hard to, at a bare minimum, send me a text about what happened.

(P2217, ACHP)

Additionally, being left out of communication about animals under their care often left residual negative feelings. One ACHP stated:

My experiences have sometimes made me feel that our hands are tied. Decisions are made from other departments; many times, the medical team disagrees, yet we aren’t always heard or listened to. It does make me feel defeated and definitely leaves a bit of contention hanging in my mind against those departments.

(P1700, ACHP)

Although many volunteers described open communication from volunteer leadership and from communication teams, others portrayed experiences of being left out of communication: “Some of us have been working with a particular animal for DECADES. Volunteers are always treated like they don’t need to know anything” (P520, Volunteer). Many volunteers expressed a desire for increased communication to support their processing of the loss or death of animals with whom they had developed strong bonds.

Those who received clear communication reported feeling relief and a greater sense of understanding. In particular, collaboration with veterinary staff appeared to foster a sense of closure for some zoo professionals as they navigated processing an animal’s death:

Frequently, our vet staff is supportive in that they allow for staff to participate in necropsies, or they give detailed information on the gross pathology results, which can help people [to] feel better about the decision to euthanize an animal, or explain why an animal may have died unexpectedly.

(P339, ACHP)

A nuanced aspect of communication discussed among some participants was their zoos’ processes for external communication. Many participants yearned for policies that would allow them to share about animal losses with zoo visitors and via personal social media:

I want to at least talk about my loss on my own freaking social media. Letting us mourn publicly and showing tribute to them in a way that honors [the animal].

(P548, ACHP)

The option to share about their loss could help some zoo professionals and volunteers with feelings of disenfranchised grief:

Recently, with new management, we have begun to publicly and internally post about some animals when they pass. I know it’s not great when every Facebook post is [about] an animal dying, but being able to share how special that animal was is so healing. They all had unique personalities, and they were all individuals that mattered and that we cared about so much.

(P2125, ACHP)

In addition, the participants noted that some visitors form connections with animals and may want to honor or remember an animal that died:

For some of the large animals that are well known to a lot of zoo visitors, I think it would be beneficial to let the community grieve as well. In the past, kids and/or other visitors have left flowers for animals or pictures at the exhibits, and our management wants us to take them down. It hurts the heart when we feel that we [and visitors] are not allowed to grieve.

(P224, ACHP)

The participants also noted that being able to externally share their loss could help them to better communicate with visitors and to be able to answer questions about why an animal is not visible:

Our zoo typically refuses to publicly acknowledge animal deaths, apart from some of the more important or recognizable individuals. This makes it incredibly difficult to grieve that loss, as it cannot be done publicly or shared on social media, and we often end up getting directly asked by guests where that animal is or what happened to them, which is then incredibly painful to answer that question. Sharing about all deaths, even in just the form of a monthly newsletter or by posting a sign out front, would allow for us to be able to grieve and process without having to act like it never happened.

(P473, ACHP)

Those who experienced effective external communication at their organizations appreciated the opportunity to express their grief openly:

I feel well supported at my institution. Most deaths are publicly acknowledged and eulogized, which is very helpful. It is healing to see comments on those social media posts from zoo visitors saying how our animals affected them over the years. The commenters also often include photos they have taken of or with the animals, which is sweet.

(P1018, ACHP)

In addition, some participants noted that open communication, before and after losses, helped them to perform better in their roles, feel supported, and promoted healing. An ACHP shared that open dialogs between zookeepers and vets about the cause of death were beneficial in their ability to find closure:

One thing my zoo does really well is allow very open communication directly between keepers and vets. I think this is critical and allows for the best possible care of the animals. At zoos where keepers and vets do not communicate directly, information can get lost. In regard to grief and loss, I think knowing as much as possible about the animals’ health and cause of death can help with closure and healing.

(P1244, ACHP)

The responses from the participants suggested that effective communication was one way to increase staff and volunteer wellbeing and improve their ability to attend to the wellbeing of animals under their care.

#### 3.2.2. Leadership Responses

The analysis of the participants’ responses suggests that how leadership reacts is a critical component in determining how zoo professionals and volunteers experience a loss. Leaders included direct supervisors, middle management, and people in the roles of assistant curators, curators, directors, executives, and top executives. Throughout the narratives, the participants called attention to favorable and less-favorable reactions from leaders. In general, it was important for leadership to acknowledge and validate losses. For instance, one person shared,

At this point, the bar seems [to be] so low; we would appreciate even the administration acknowledging that the death of an animal can have a significant impact on keepers. Most of the time, they take the news as if we lost a set of keys and have little to no empathy for keepers.

(P1720, ACHP)

Another important consideration for many of the participants was how leaders and organizations failed to equitably value the life and death of animals (and those who care for them) across taxa. Some participants commented that when their organization provides support for keepers, it is usually after the loss of species that might be considered as large, charismatic megafaunae, such as great apes, large cats, or elephants. Many participants shared that other species (and their keepers) do not receive the same recognition or support. This commonly resulted in feelings that one’s organization did not value species such as birds, reptiles, fish, amphibians, and other smaller animals:

When large mammals or carnivores pass, the zoo [communications] will make a post about it or make some comment about it. However, I’m a bird and small-hoofstock keeper, so my animals normally don’t get any recognition. I’m aware that birds die much easier than, say, a tiger, but they are still animals that we care for and bond with, and when you hear upper management, and even other keepers, say, ‘It’s just a bird’, it is very disheartening.

(P1885, ACHP)

Specific suggestions for individual leadership and organizational leadership included showing empathy to zoo professionals and volunteers following a loss, recognizing and validating the feelings of the staff, and reaching out proactively to check in with the staff after a loss.

## 4. Discussion

The findings from this study present a compelling narrative for understanding how U.S. zoo professionals and volunteers experience animal death and transfer. Building on findings about zoo professional burnout and loss [53], this study presents descriptive examples of the immediate and long-lasting effects of zoo animal loss. Of note, the quotes shared in the results section portray the mild, less-graphic experiences shared by the participants. Whether through an animal’s death or transfer, these losses create immediate and long-lasting effects on the mental health of zoo professionals and volunteers as well as compromise their abilities to conduct daily animal care tasks and maintain both human and animal safety.

Although some zoo professionals and volunteers described supportive peer and leadership responses, the majority of the participants in this study portrayed zoo cultures with limited organizational structures to ritualize or openly communicate about loss, inequitable provisions of grief resources and support, and distressing interpersonal responses from leadership. Significant losses, followed by the limited provision of grief support, left many zoo professionals and volunteers feeling isolated in their grief, facing unsupported shame, guilt, numbness, and, for some zoo professionals, dissociation. Rituals surrounding zoo animal loss may foster a sense of community and opportunities for continuing bond expression and reduce zoo professionals’ experiences of anxiety and depression [36].

The zoo professionals and volunteers shared stories of attachments they developed with animals at the zoo, whether as a part of their role in daily care, as a volunteer interacting with the public through ambassador animals, or as someone who works in less-hands-on roles with the animals. They expressed gratitude for being able to build these relationships with these animals and detailed the care and work they went through to ensure that the animals lived in positive environments. Mirroring previous research findings [42,43,44,45,46], the respondents felt an onus of responsibility for ensuring the livelihoods of endangered species. As such, the level of the care provided and depth of the bond served to augment the emotional turmoil experienced when an animal died [47,48,49]. If traumatic events surrounded the death, these losses came with additional layers of questioning personal actions, guilt, and shame. For example, not advocating to end what appeared to be futile care resulted in expressions of regret and interpersonal frustration. Euthanasia procedures offered some relief, knowing that animals would no longer suffer, and created distressing memories when procedures were extended or did not go as planned [37,38,39,40,41].

Organizational practices, such as limited staffing, lack of transparency, and impersonal or insensitive interactions with colleagues, served to increase feelings of despair and extended the time individuals spent processing their grief. Many individuals had to quickly return to animal care activities or interactions with guests because of staffing concerns and programming expectations. These abrupt shifts from witnessing deaths to returning to work activities resulted in some ACHPs feeling that they had to emotionally close off to perform their roles [47,48]. Emotional distancing can serve as a coping mechanism to distance from work-related death and trauma; however, the long-term practice of distancing may result in complicated grief [67], emotional exhaustion [68], and compassion fatigue [36].

Continued exposure to death and transfer losses among zoo professionals and volunteers [47,52,53] can foster a cycle of attachment and loss, which, without dedicated psychosocial support and resources, may result in complicated grief responses [36,51]. As evidenced in the narratives of the participants’ emotional numbness, burnout, and dissociation, as well as earlier research, data suggest that distressing mental health and wellbeing already exist within zoo professionals [36]. Of concern, research indicates that unresolved trauma and grief may lead to an increased risk for the development of posttraumatic stress disorder [69,70]. Future research should extend this study’s findings to explore experiences of posttraumatic stress disorder among zoo professionals and volunteers, resulting from their exposure to zoo animal loss.

Building on findings of negative sequelae from pet loss research [18,19,20,21], this study highlights the disenfranchised nature of zoo animal loss. Zoo professionals and volunteers elevated the disenfranchised nature of transfer loss and the need to recognize this type of loss as significant. Their experiences are akin to those of service dog owners who grieve the relationship of their service dog when the dog is rehomed for retirement [71,72]. Similar to pet loss, specialized resources available for grieving zoo professionals are limited, highlighting the importance for developing tailored support mechanisms within the zoo industry. Notably, stories of positive internal and external support shed light on existing grief support activities in some zoos. Examples include departmental peer support activities (both informal and formal), the sharing of tasks following a death, rituals to memorialize animals who have died or been transferred, and validation by friends working at other zoos, who reached out to send their condolences.

Echoing throughout the participants’ responses was an understanding that everyone copes with zoo animal death and transfer losses differently. No one solution will work across all zoo cultures. However, it was clear that resources to foster open dialog about loss, both pre- and post-death and transfer, are seen as vital to the sustainability of zoo professionals’ and volunteers’ wellbeing. Creating cultures of transparency, where individuals can openly discuss pending losses and understand the circumstances surrounding death, may also foster willingness to seek support and enhance interpersonal trust. Creating opportunities for the development of policies to ritualize losses and increase community support around grief may help to reduce experiences of disenfranchised grief and promote wellbeing among zoo professionals, volunteers, and zoo animals [52,63].

### Limitations/Future Directions

A few limitations to this study bear mentioning. The study was available to zoo professionals and volunteers across the United States through an extensive recruitment process. However, it is possible that some individuals did not receive a notice about the study or were not comfortable in sharing about their loss experiences within their work or volunteer grief setting. The sample consisted of predominantly white cisgender women, and themes emerging in this sample may not reflect staff of other gender identities or those from marginalized racial, ethnic, language, or cultural groups. We asked the participants to share about their most significant death experience, and some chose a recent event, while others chose losses that happened several years ago, and recollection bias may be of concern. However, the feelings expressed and detailed descriptions of the events demonstrated the long-lasting impacts of the losses, despite the extended time since the events. Notably, people may have transitioned roles or zoo locations, and the events portrayed may represent experiences at former facilities.

In the current study, animal transfers were characterized as a type of loss. However, animal transfers may not be considered as losses by all the zoo professionals or volunteers. For example, some animal keepers may hold positions where their primary role is to breed animals for other zoos. Although keepers may be attached to the animal, the ability to move animals to other zoos for the purpose of species survival or recovery may be considered as a professional and programmatic success, one that could be celebrated as a demonstrated testament to the quality of the care provided by zoo professionals. In other cases, animal transfers may provide opportunities for an animal to be moved to a better social situation or as a part of a species survival plan, thus not being perceived as a loss by zoo staff or volunteers. Therefore, transfers can also be events that keepers can take pride in and may be associated with a sense of professional fulfillment and positive emotions. Future research pertaining to transfers, given their unique type of loss and potential fulfillment, is warranted.

Additional avenues of future research could be conducted to help to elucidate the specific types of experiences that generate greater feelings of grief and loss and potentially negatively impact zoo professionals’ and volunteers’ quality of life and professional wellbeing. It will also be important to evaluate the supportive services and interventions implemented to ensure that they are having the desired effects.

This research, together with previous studies, suggests that animal loss, through both death and transfer, can negatively impact zoo professionals and volunteers’ wellbeing and psychological health. This knowledge, at the individual micro level, helps to create the foundation needed to explore systemic, macro-level interventions to better support zoo professionals and volunteers and enhance the welfare of the animals under their care.

## 5. Conclusions

Although exposure to animal loss appears to be an occupation-related certainty among zoo professionals and volunteers, individuals describe the support they received both pre- and post-loss as oftentimes less than what is needed. Participants long for ritual, transparency, communication, and empathy, as well as resources to prepare for and adapt to animal transfers and deaths. The participants’ narratives highlight the heavy emotional burden felt by many following an animal loss and shed light on how some individuals utilize numbing and dissociation to cope with their grief. These findings call attention to the dire need for zoo cultures to foster environments where professionals and volunteers can find community in the experience of and adaptation to zoo animal loss.

## Data Availability

The data are available upon request.
